# Discovery of a mutation-containing circRNA in polyglutamine disease through systematic analysis of RNAs with CAG repeats

**DOI:** 10.1080/15476286.2026.2684791

**Published:** 2026-06-24

**Authors:** Weronika Pawlik, Magdalena Woźna-Wysocka, Magdalena Jazurek-Ciesiołka, Jarosław Dulski, Tomasz M. Witkoś, Agata Ciołak, Emilia Kozłowska, Edyta Kościańska, Luke C. Bartelt, Julien Philippe, Jarosław Sławek, Paweł M. Świtoński, Albert R. La Spada, Agnieszka Fiszer

**Affiliations:** a Department of Medical Biotechnology, Institute of Bioorganic Chemistry Polish Academy of Sciences, Poznan, Poland; b Department of Neurology, Neurodegenerative Diseases and Neuroimmunology, Medical University of Gdansk, Gdansk, Poland; c Department of Molecular Genetics and Microbiology, Duke University Medical Center, Durham, NC, USA; d Departments of Pathology & Laboratory Medicine, Neurology, Biological Chemistry, and Neurobiology & Behavior, and the Center for Neurotherapeutics, University of California, Irvine, CA, USA; e Department of Neuronal Cell Biology, Institute of Bioorganic Chemistry Polish Academy of Sciences, Poznan, Poland

**Keywords:** CAG repeats, non-coding RNA, circRNA, lncRNA, polyglutamine diseases, SCA7

## Abstract

CAG repeat tracts occur in both non-coding and translated RNAs, have tended to lengthen throughout evolution, and are thought to enhance neuronal function. We identified over 600 human RNAs (including mRNAs, lncRNAs, and circRNAs) with at least 10 CAG repeats, originating from 58 genomic *loci*, which vary, e.g. in the rate of CAG length polymorphism. Several circRNAs originate from the *ATXN7* locus, where CAG expansion causes spinocerebellar ataxia type 7 (SCA7). For selected *circATXN7(3,4).1* (circ1), we demonstrated its cytoplasmic localization, as well as its presence in 40S, monosome and polysome fractions. We showed that circ1 is expressed in human fibroblasts, blood and cerebellum, and, importantly, we identified a mutation-containing circRNA with potential implications in SCA7.

## Introduction

CAG repeat tracts are examples of short tandem repeats (STRs) present in the human genome. CAG repeats are among triplet repeat tracts overrepresented in the transcriptome and are present in protein-coding and non-coding RNAs (ncRNAs) [1,2]. The length of CAG tracts generally increases with the complexity of organisms and has been associated with better brain functioning [3]. On the other hand, expansions of these sequences above the defined threshold have adverse effects and cause neurodegenerative disorders, including polyglutamine (polyQ) diseases, like Huntington’s disease (HD) and several types of spinocerebellar ataxias (SCAs) [4–6]. While the significance of CAG repeats in protein-coding sequences is well established [7,8], their prevalence and potential functional roles in non-coding RNAs remain underexplored. Various ncRNAs, including long non-coding RNAs (lncRNAs) and circular RNAs (circRNAs), have emerged as key players in diverse cellular processes, often in a cell-type- and developmental stage-dependent manner, which causes additional challenges in studying them [9,10]. lncRNAs are a highly heterogeneous group involved in chromatin remodelling, transcriptional regulation, and RNA processing [11]. circRNAs, in turn, are covalently closed transcripts with high stability and often exhibit tissue-specific expression and regulatory potential, particularly in the nervous system [12]. Here, we identified a set of RNAs that contain repeat tracts of at least 10 CAG units and present their variable features of the repeat tract sequence. We experimentally validated one of the circRNAs from the *ATXN7* locus, demonstrating an example of an ncRNA with a CAG tract and potential functional and disease implications.

## Materials and methods

### Retrieval of coding and ncRNAs with CAG tracts from databases

An in-house Python script was used to identify human RNAs containing at least 10 CAG repeats among protein-coding transcripts, lncRNAs, and circRNAs, as previously described [1]. Protein-coding transcripts and lncRNAs were retrieved from GENCODE release 49 [13], and human circular RNA datasets from circAtlas 3.0 [14]. Identified RNAs are listed in Table S1. See Supplementary Methods for more details on bioinformatics analyses performed.

### Cell lines, human samples and RNA isolation

Human fibroblast cell lines – SCA7 (GM03561) and healthy controls (GM07525, GM04503, GM07522, GM07492) (Coriell Institute), as well as HEK293T cells and neural stem cells (NSCs) were cultured in standard conditions (see Supplementary Methods for more details). The GM07522 line was reprogrammed to induced pluripotent stem cells (iPSCs), as described previously [15], and differentiated to NSCs as described previously [16].

Total RNA was isolated from cell pellets and polysome fractions with TRI Reagent (Invitrogen) using the Total RNA Zol-Out D kit (A&A Biotechnology) following the manufacturer’s protocol (see Supplementary Methods for more details). Human blood samples were collected from three symptomatic patients with SCA7 from Polish Family 1 [17] and from healthy individuals, using PAXgene Blood RNA Tubes (Qiagen). Total RNA was isolated from peripheral whole blood using the manual protocol of the PAXgene Blood RNA Kit (PreAnalytiX), according to the manufacturer’s instructions. See more details and the RNase R treatment protocol in Supplementary Methods.

Human post-mortem cerebellar tissues were obtained from the University of Florida Center for NeuroGenetics (*n* = 5, SCA7 samples), the University of Michigan Brain Bank (*n* = 3, healthy control samples) and UCI-ADRC (*n* = 2, healthy control samples). RNA was purified with the Direct-zol RNA MiniPrep Plus kit (Zymo Research) or RNeasy Plus Kit (Qiagen). See Supplementary Methods for more details.

### Nuclear and cytoplasmic fractionation

Fibroblast cells were fractionated using NE-PER Nuclear and Cytoplasmic Extraction Reagents (Thermo Scientific) according to the manufacturer’s protocol. See Supplementary Methods for more details.

### Reverse transcription and qPCR

cDNA was synthesized from 0.5 µg RNA for cells, blood samples and RNA after RNase R treatment using SuperScript IV Reverse Transcriptase (Thermo Fisher) with random primers, according to the manufacturer’s protocol. The resulting cDNA was then diluted 1:10 and 2.5 µL was used with the SsoAdvanced Universal SYBR Green Supermix (BioRad) and run on a CFX Connect system (BioRad). For human cerebellum samples, cDNA was reverse transcribed from 0.5 µg RNA with SuperScript IV (Thermo Fisher) using random hexamers. An input of 1 µL (~6 ng) of diluted cDNA (1:5) was used with the Thermo PowerTrack SYBR Green mastermix for RT-qPCR in a 10 µL reaction volume in 384-well plate. Each target was run in triplicate, with a no-RT control, and Cq values were measured on a QuantStudio 6 instrument (Applied Biosystems).

### Primer design

Primers were designed using the NCBI Primer-BLAST tool with standard parameters applied. Binding sites for primers at the *ATXN7* locus are shown in Figure S1. All primers were tested for amplification efficiency and specificity before use. Primer sequences are listed in Table S2.

### Back-splice junction confirmation and CAG tract assessment

RT-PCRs were performed using GoTaq Flexi DNA Polymerase (Promega) with 1 M betaine and run on a T100 Thermal Cycler (BioRad). Products were resolved on 1.5% agarose gels, purified using PureLink Quick Gel Extraction Kit (Invitrogen), and sequenced by Sanger sequencing. For RT-PCR products obtained from human cerebellum samples, Premium PCR Sequencing (Plasmidsaurus) was performed by using Oxford Nanopore Technology with custom analysis and annotation.

### Polysome profiling

Polysome profiling was performed as described previously [18]. More details and RNA isolation from fractions is described in Supplementary Methods. cDNA was synthesized from an equal volume of RNA from the polysome fraction using High-Capacity cDNA Reverse Transcription Kit (Applied Biosystems) with random primers, according to the manufacturer’s protocol. Reactions were prepared using the SsoAdvanced Universal SYBR Green Supermix (BioRad) and run on a CFX Connect system (BioRad). Each RNA sample was assayed in triplicate.

### Statistical analysis

Statistical analyses were performed in GraphPad Prism. The level of significance (alpha) was always set at 0.05. Only statistically significant results are marked in the figures with *p < 0.05and **p < 0.01.

## Results

### Tracts of ≥10 CAG repeats are present in various RNA types and show diverse features

**Figure 1.**
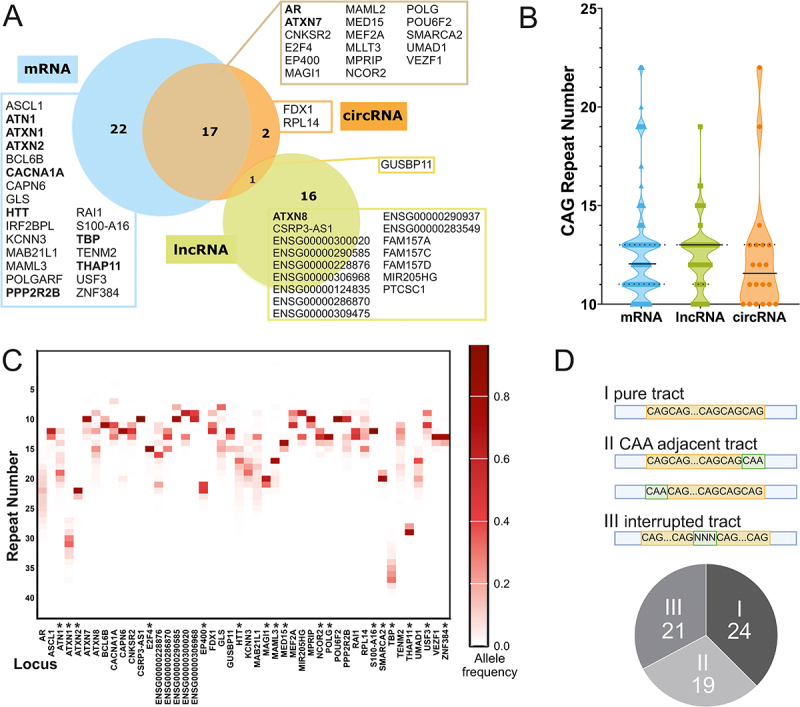
RNAs with at least 10 CAG repeats: biotypes, tract polymorphism, and sequence patterns.

In this study, we focused on RNAs with at least 10 CAG repeats, as such sequences are more likely to have a variable length in the population and/or to fulfill a functional role in the molecule. Previous studies explored the occurrence of shorter CAG tracts, i.e. composed of at least 5 or 6 units [1,2,8,19]. Over time, the number of annotated CAG-containing RNAs increased [13]; therefore, in this study, a threshold of 10 CAG repeats was sufficient for the exploration of numerous molecules (and with a higher probability of CAG tract importance for the molecule). Using the reference datasets, we identified a set of human RNAs, including 423 mRNAs, 147 lncRNAs, and 93 circRNAs (Fig. S2, Table S1) originating from 58 genomic loci. Specifically, the identified mRNA, lncRNA, and circRNA sequences originated from 39, 17, and 20 genomic loci, respectively (Figure 1(A)), with highly variable numbers of transcript variants per locus (Fig. S2). Identified loci include genes involved in transcription (*E2F4*, *IRF2BPL*), Alu-mediated gene expression regulation (*MIR205HG*), neuronal function (*HTT*, *ATXN2*), and chromatin remodelling (*SMARCA2*, *EP400*). Several identified RNAs are transcribed from genes with CAG tract mutations described, mainly those implicated in polyQ diseases [6]. From most identified loci (exactly 39), mRNAs are produced, indicating that ≥10 CAG repeat sequences are predominantly associated with protein-coding genes. A considerable number of CAG repeat-bearing transcripts (exactly 17) is classified as lncRNAs, with only one locus that also produces circRNA (Figure 1(A)). Also, a substantial number of loci (i.e. 17) produce both mRNAs and circRNAs, suggesting that many CAG-containing exons in mRNAs undergo back-splicing, producing circular isoforms. Among these loci, *CNKSR2* and *UMAD1* are the only ones that have repeats located in the UTR of corresponding mRNAs. Other circRNAs originate specifically from exons present in coding regions in mRNAs. Analysis revealed many circRNAs have a repeat tract in close proximity to the BSJ (≤50 nt) (Fig. S3), which could potentially affect back-splicing in case of variable CAG tract length in the population, especially in case of repeat expansion. Interestingly, a few *loci* — *CNKSR2*, *MAGI1*, and *UMAD1* harbour repeats in less than 10 nt distance to the BSJ.

(A) Venn diagram of 58 genomic *loci* with ≥10 CAG repeats, showing the classification of molecules: mRNA (blue), lncRNA (green), and circRNA (orange), with indicated *loci* that produce two different types of RNAs. Bolded gene names represent *loci* known to be associated with repeat expansion disorders.

(B) Distribution of CAG tract lengths in different RNA biotypes (based on reference sequence in databases). Violin plots show kernel density estimation of the distribution of CAG tract length in mRNAs (blue), lncRNAs (green), and circRNAs (orange). Each symbol represents an individual RNA molecule. Horizontal lines within the violins represent the median values; horizontal dashed lines represent quartiles.

(C) Heatmap showing allele frequency distributions of repeat lengths across CAG-containing *loci*, based on data deposited in TR-Atlas (338,963 human samples in diverse ancestries; data were available for 46 of the analyzed *loci*). Repeat number refers also to other triplets, as not all tracts are pure CAG (*loci* with interruptions are marked with an asterisk).

(D) Classification of CAG tract types and pie chart with the number of*loci* in each category. All tracts contained at least 10 CAG repeats and were classified as: (I) pure, (II) with adjacent CAA triplet (at 3’ or 5’ site), or (III) interrupted (containing at least one trinucleotide different from CAG, that precedes or follows a tract of at least 10 pure CAG repeats, and is also preceded or followed by at least one CAG trinucleotide). A total of 64 tracts were classified due to the presence of tracts that have both a CAA adjacent codon and an interruption in the*IRF2BPL, MAML2, MED15, SMARCA2, USF3* and *ENSG00000306968 loci*.

Analysis of reference database sequences showed that most identified RNAs contained 10–15 CAG repeats, although longer tracts were observed across all three RNA classes (Figure 1(B), no statistically significant differences in CAG tract lengths were found between RNA types). Recent advancements in the analysis of polymorphism of repeat tracts in populations [20,21] enabled us to analyse selected CAG-containing loci in this aspect. Due to sequence/structure-related aspects and/or selective pressure favouring specific repeat lengths, the CAG tracts at individual loci vary significantly in length range within the population. Some of the loci, like *CSRP3-AS1*, *POU6F2*, and *S100-A16*, show the dominance of a single-length variant, whereas other loci, like *AR*, *HTT*, and *MAB21L1*, are characterized by a large variation of the number of CAG units [21] (Figure 1(C)).

We classified the CAG repeat tracts in identified loci as: (I) pure, (II) with adjacent CAA, or (III) interrupted (Figure 1(D)). Many loci (i.e. 24), such as *ATXN7*, *CACNA1A*, and *PPP2R2B*, contain uninterrupted CAG repeats. These pure tracts are typically more prone to expansion, increasing the risk of disorders like SCAs. Other loci contain interruptions within the CAG repeat, which can be single, as in *ATXN2* and *HTT*, or multiple, as for *SMARCA2* and *TBP*. These interruptions can influence the DNA and RNA structure of the repeat tract [22,23], reducing expansion rates and delaying disease onset in the case of a mutation [24–26]. There were 19 loci, including *AR* and *MIR205HG*, with CAA triplets adjacent to CAG tracts. CAA, like CAG, encodes glutamine, thereby leading to a longer polyQ tract for repeats present in the translated region.

### Circ1 from the ATXN7 locus shows cytosolic localization and ribosome association

Among the loci identified in our analysis, the *ATXN7* locus was prioritized for further investigation due to its direct relevance to repeat expansion disorders and the presence of multiple CAG-containing circRNA isoforms, making it a particularly suitable model for focused validation. In this locus, we identified nine circRNAs that contained a CAG repeat tract (Fig. S4A). The expansion of the CAG tract in the *ATXN7* gene, which encodes the ataxin-7 protein (containing an expanded polyQ tract when mutated), causes SCA type 7 (SCA7) [4]. CircRNAs identified at the *ATXN7* locus, which were formed from various exon combinations, ranged in length from 336 to 1106 nt and all contained the entire exon 3 of *ATXN7* mRNA (Fig. S4A). For further experimental validation, we selected hsa-ATXN7_0001 (circATXN7(3,4).1; hereafter ‘circ1’), based on the substantial expression levels of this circRNA in human brain and blood [14] (Fig. S4B). Moreover, circ1 showed a higher ‘circular to linear’ ratio in the brain as compared to the liver, while the linear form of *ATXN7* RNA showed a slightly lower level in the brain than in the liver (Fig. S4C).

**Figure 2.**
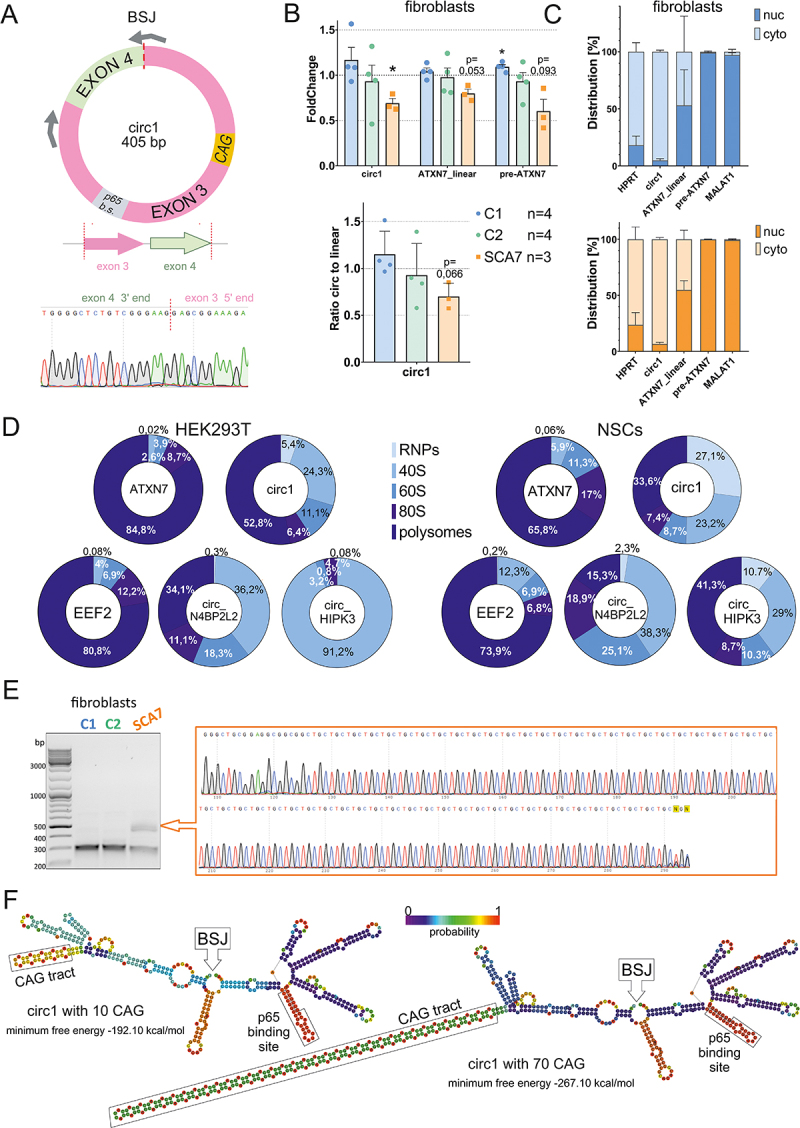
Experimental validation of *ATXN7* circRNA and mutant variant identification in SCA7 fibroblasts.

To confirm the circular nature of circ1, we sequenced its back-splice junction (BSJ) in PCR products from brain tissue (Figure 2(A)) and fibroblasts (Fig. S5A), as well as performed circ1 and *ATXN7* mRNA amplification following RNase R treatment (Fig. S5B, C). Next, the levels of circ1 and linear ATXN7 RNA were assessed in human fibroblasts (Figure 2(B) and S1). We observed a statistically significant reduction in circ1 expression in SCA7 fibroblasts, to ~70% of the level in the control cell lines (Figure 2(B), upper graph). The linear *ATXN7* RNA showed a tendency for modest reduction (to ~ 80%) in the SCA7 line compared to the control lines. As a consequence, a trend towards a lower circular to linear ratio was observed for circ1 in the SCA7 line (Figure 2(B), lower graph), suggesting that biogenesis of this circRNA may be altered in SCA7. Moreover, based on the results of *ATXN7* pre-mRNA level, there is a tendency (*p* = 0.09) for already lower transcription from *ATXN7* locus in SCA7 fibroblasts, as compared to control lines (Figure 2(B), upper graph).

(A) Schematic representation of circ1 (hsa-ATXN7_0001; Uniform ID: circATXN7(3,4).1) with exon composition below and Sanger sequencing results from a human brain sample, showing back-splice junction (BSJ), indicated by dashed red line. Grey arrows indicate the location of divergent primers used for BSJ site sequencing.

(B) The relative level of circ1, *ATXN7* mRNA (ATXN7_linear) and pre-mRNA (pre-ATXN7) in human fibroblasts was assessed using RT-qPCR. C1 (GM07525; *n* = 4), C2 (GM04503; *n* = 4) – healthy individuals; SCA7 (GM03561; *n* = 3) – patient’s line, where n is the number of independent sample collections. For circ1 expression analysis, we used primers that spanned the specific BSJ sites (but not the CAG tract) and obtained single products of the expected length. The mean result from the control lines was set as 1 for normalization. As a reference, the expression levels of *HIPK3* (circHIPK3(2).1) and *N4BP2L2* (circN4BP2L2(3,4,5S,S6).2) were used for circRNA, and *GAPDH* and *EEF2* for linear RNA. The circ/linear RNA ratio was calculated using linear RNA Ct as a reference; values are presented as fold changes over linear RNA. Statistical significance was determined by a one-sample t-test with a hypothetical mean = 1. *p < 0.05

(C) The distribution of circ1, mRNA and pre-mRNA of *ATXN7* transcripts in the fibroblast cell lines assessed by nucleus-cytosol fractionation. Blue graph (upper) shows the distribution in C1 (healthy cell line; *n* = 3) and orange graph (lower) – in SCA7 (patient’s line; *n* = 3), where n represents the number of independent sample collections.

(D) Doughnut plots visualizing distribution of circ1 and*ATXN7*in fractions of ribonucleoproteins (RNPs), 40S, 60S, 80S and polysomes in HEK293T cells (left) and NSCs (right) with linear (*EEF2*) and circular (*HIPK3* and *N4BP2L2)* reference RNAs shown below.

(E) RT-PCR products (containing CAG repeat tract and BSJ site) for*circ1* obtained from RNA isolated from human fibroblasts (C1, C2 – healthy individuals; SCA7 – patient’s line). The slower-migrating band obtained from the SCA7 sample was sequenced and confirmed to contain an expanded CAG tract.

(F) Predicted secondary structures formed by *circ1* with 10 CAG (wild-type variant) and 70 CAG (mutation in SCA7) using RNAfold. The CAG region, BSJ site, and NF-kappaB p65 binding motif are indicated in both structures. The colour scale shows predicted probabilities for pairing or remaining unpaired for each base.

To gain further insight into potential circ1 functions, we investigated its subcellular localization, together with *ATXN7* mRNA and pre-mRNA in fibroblasts. In both control and SCA7 cell lines, we observed similar and expected distribution of *HPRT* mRNA and *MALAT1* lncRNA, i.e. preferential cytoplasmic and nearly exclusive nuclear localization, respectively, confirming proper fractionation (Figure 2(C)). In both cell lines, *ATXN7* pre-mRNA showed the expected nuclear localization, whereas *ATXN7* mRNA showed nearly equal distribution between both fractions. On the other hand, almost all circ1 was localized in the cytosol in both cell lines (Figure 2(C)). Given its predominantly cytosolic localization, we next examined whether circ1 could be translated or play a role in translation regulation. According to the riboCIRC database [27], circ1 possesses IRES sequence [28] and potential ORF, resulting in ~ 14 kDa peptide containing CAG-encoded polyserine (polyS) tract (Fig. S6). To obtain experimental insights, we performed polysome profiling of lysates from HEK293T and neural stem cells (NSCs) and determined the circ1 co-sedimentation profile with ribosome subunits and polysomes (Fig. S7). In both cell lines, *ATXN7* mRNA showed an expected association with translation machinery, similar to reference *EEF2* mRNA (more than 80% of mRNAs were detected in 80S and polysomes fractions) (Figure 2(D)). For all analysed circRNAs (circ1 and reference circRNAs: *HIPK3* and *N4BP2L2*), we observed variable profiles that may be related to their functions. Circ1 showed substantial co-sedimentation with polysomes, i.e. ~53% of circ1 in HEK293T cells and ~34% in NSCs. Circ1 was also present at substantial levels in 40S and 60S fractions. Moreover, in NSCs, we observed enrichment of circ1 in the free cytosolic light components fraction (ribonucleoproteins, RNPs). As circ1 co-sedimentation profiles differ between HEK293T cells and NSCs (with a similar observation for *HIPK3* circRNA), this indicates cell-type-specific specialization of circRNAs. Our results suggest that circ1 may be translated or play a role in translation, based on its association with the 40S ribosomal subunit.

### Expanded CAG tract in circ1 is present in SCA7 patient cell line, blood and cerebellum samples

To determine whether circ1 contains a CAG repeat expansion in SCA7 cells, we designed primers spanning both the CAG tract and the BSJ site. RT-PCR on RNA isolated from SCA7 fibroblasts revealed the presence of both a normal and an expanded CAG tract (with at least 56 repeats) within circ1 (Figure 2(E)). This constitutes the first described case of a circRNA containing a mutation in the form of a repeat expansion. To gain further insight into the potential effects of the mutation, we modelled the secondary structure of circ1 (Figure 2(F)) [29]. We found no apparent differences between circ1 with 10 or 70 CAG repeats in the regions spanning the BSJ site. Interestingly, circ1 also contains the NF-kappaB p65 subunit binding motif [30] that was still present in both structures (Figure 2(F)). As expected, the expanded CAG tract formed an elongated hairpin structure, which caused higher predicted stability of circ1 secondary structure with the mutant CAG tract, as compared to circ1 with the normal CAG tract (based on calculated minimum free energy, Figure 2(F)).

To further characterize the potential regulatory interactions of circ1, predicted miRNA binding sites were mapped along the circRNA sequence based on seed complementarity (Fig. S8(A), Table S3). A total of 174 binding sites were identified for 35 miRNAs, with 8mer, 7mer-m8, 7mer-A1 and 6mer seed matches represented. Similarly, predicted RBP binding motifs retrieved from the ATtRACT database [31] were mapped along the circ1 sequence (Fig. S8(B), Table S4). Binding motifs were identified for 7 RBPs, with SRSF1, PTBP1, and KHSRP displaying the highest number of predicted binding sites.

**Figure 3.**
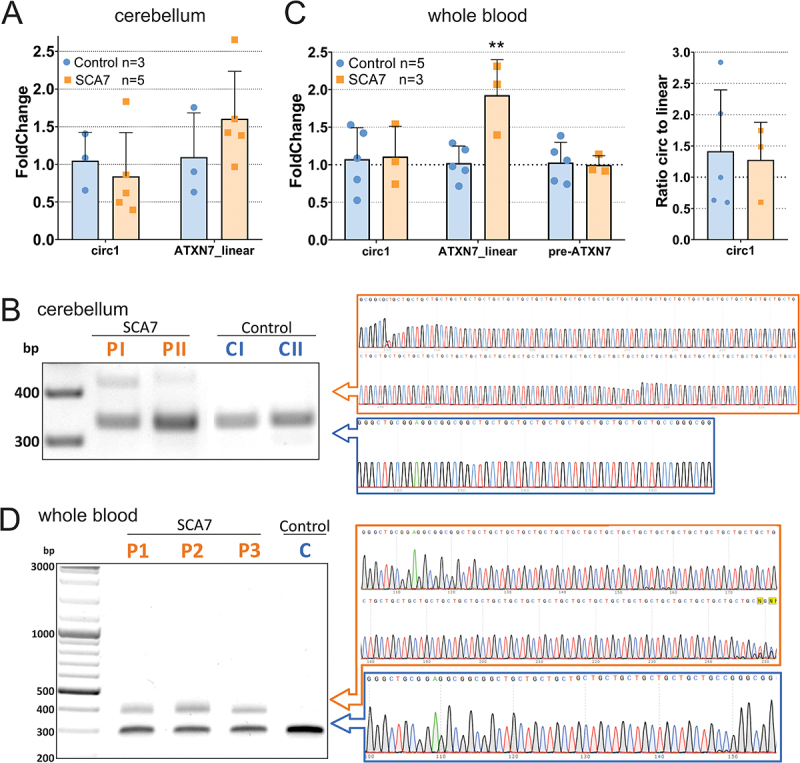
Expression of *ATXN7* circRNA in human tissues and mutant variant identification in SCA7 patients’ cerebellum and blood.

Given that the cerebellum is major site of SCA7 neurodegeneration, we investigated circ1 expression and detected reads supporting circ1 presence in human foetal cerebellum datasets [32,33]. Next, we experimentally analysed the circ1 level in adult human cerebellum tissue obtained from healthy individuals and SCA7 patients and detected it in all samples. No statistically significant difference was observed in circ1 or linear *ATXN7* RNA levels between control and SCA7 samples (the variability between samples was relatively high) (Figure 3(A)). Moreover, we detected circ1 with a mutant CAG tract (containing 53 and 57 repeats) in two of the SCA7 patient cerebellum samples (Figure 3(B)).

(A) The relative level of circ1 and *ATXN7* mRNA was assessed using RT-qPCR in human cerebellum samples from healthy individuals (control, *n* = 3) and SCA7 patients (*n* = 5), where n is the number of individuals. For normalization, the mean result from control lines was set as 1. As a reference, the expression levels of *HIPK3* and *N4BP2L2* were used for circRNA, and *GAPDH* and *EEF2* for linear RNA. Statistical significance was determined by an unpaired t-test.

(B) RT-PCR products of circ1 containing CAG repeat tract and BSJ site, obtained from human cerebellum samples (CI, CII – healthy individuals; PI, PII – SCA7 patients). The slower migrating bands were confirmed to contain an expanded CAG tract.

(C) The relative level of circ1, *ATXN7* mRNA and pre-mRNA was assessed using RT-qPCR in human whole blood samples from healthy individuals (control, *n* = 5) and SCA7 patients (*n* = 3), where n is the number of individuals. Normalization, reference RNAs and statistics – as described in B. The circ/linear RNA ratio was calculated using linear RNA Ct as a reference; values are presented as fold changes over linear RNA. Statistical significance was determined by a one-sample t-test with a hypothetical mean = 1. *p*-values less than 0.05 were considered significant. **p < 0.01

(D) RT-PCR products of circ1 containing CAG repeat tract and BSJ site, obtained from human blood samples (C – healthy individual; P1, P2, P3 – SCA7 patients). Indicated products were sequenced. The slower migrating bands were confirmed to contain an expanded CAG tract.

Next, to refer to a potential investigation into the disease course and high expression level of circ1 in blood (Fig. S4(B)) [14], we analysed blood samples from three symptomatic SCA7 patients [17] and detected circ1 in all cases. RNase R digestion confirmed circularity of circ1 (Fig. S9). We found no apparent differences in the levels of circ1, but the linear *ATXN7* RNA showed an increased level (~2-fold) in SCA7 patients’ samples, as compared to healthy individuals (Figure 3(C)). Moreover, circ1 with a mutant CAG tract (containing >42 repeats) was also detected in samples from all three SCA7 patients (Figure 3(D)).

## Discussion

As ncRNAs are generally known to have higher expression and specific functions in the brain, their investigation is an emerging field in molecular neurobiology [34,35]. Furthermore, for neurodegenerative diseases, where a key protein component has been identified, it becomes essential to investigate ncRNAs that may significantly contribute to altered pathways and serve as biomarkers, given their high stability and presence in extracellular vesicles (EVs), as seen for circRNAs [12]. For example, a recently described circRNA from the *HTT* locus, but lacking the CAG repeat region was described as a modifier of the HD phenotype [36,37].

To the best of our knowledge, the presence of the CAG tract has not yet been explored in ncRNAs. We show that 36 loci produce ncRNAs that contain CAG tracts. Some of these tracts exhibit length variability in the population, which could affect ncRNA function independently of expression levels. The CAG tract length in the *ATXN7* locus is not highly polymorphic in the population [20,21] with ~75% people carrying 10 CAG repeats in *ATXN7*, and ~12% carrying 12 CAGs [21] (Figure 1(C)). Interestingly, circ1 is one of the most abundant from the *ATXN7* locus (Fig. S4B) and is predicted to bind miRNAs and proteins (Fig. S8) [14]. This way, a long CAG tract in mutant circ1 may trigger adverse effects due to RNA toxicity pathways [38,39], e.g. could serve as a potent binding site for sequestering of proteins [40,41]. Although the exact sequence of circ1 is also present in linear *ATXN7* mRNA, potential interactions of this circRNA are expected to be more prominent than for the linear counterpart, due to greater access caused by the lack of canonical translation. Interestingly, in our previous study, 6 miRNAs were identified that potentially could bind to the CAG tract using 6-mer binding with the seed sequence [1], and this interaction could have more implications in the case of an expanded CAG tract. What is more, exon 3 of *ATXN7*, which gives rise to circ1, also contains an experimentally validated binding site for the p65 subunit of NF-kappaB [30] (Figure 2(F)). In cancer, elevated levels, together with cytoplasmic sequestration of p65 and reduced NF-kappaB transcriptional activity, were reported [30]. Moreover, NF-kappaB dysfunction was documented in SCA7, although it was attributed to proteasome inhibition by mutant ataxin-7 [42]. Whether additional pathways contribute to NF-kappaB dysfunction in SCA7 remains to be investigated. Our cellular localization results suggest that *ATXN7* mRNA, rather than circ1, is more likely to interact with p65 in the nucleus, at least in human fibroblasts (Figure 2(C)). Potentially, circ1 could sequester p65 to the cytoplasm.

Based on our observations, the cytoplasmic activity of circ1 is associated with the translation machinery (Figure 2(D)), which is a relatively common observation for circRNAs [43]. Circ1 may undergo IRES-dependent translation, which, based on performed predictions (Fig. S6), would result in an expanded polyS tract in the peptide produced from mutant circ1. Alternatively, repeat-associated non-AUG (RAN) translation could be initiated and lead to the production of toxic polyQ, polyS or polyalanine (polyA) peptides [44,45]. Overall, multiple adverse pathways could be induced by the presence of an expanded CAG tract in circ1, and therefore, investigating the localization and interactions of circ1 in neurons will be important.

For circ1, we confirmed the presence of a mutant variant in SCA7 fibroblasts, as well as in cerebellum and blood from SCA7 patients. Based on the obtained circular/linear ratios, the biogenesis of this circRNA was not largely affected by the mutant CAG tract, that is located 98 nt distance from BSJ. Interestingly, mutant CAG repeats were shown to act as splice acceptor sites and cause alterations in transcript variants generated from mutation-containing loci [46]. Therefore, potential alterations of *ATXN7* splicing should be investigated in more detail.

In summary, our findings expand the current view of repeat-containing ncRNAs as previously underappreciated carriers of CAG tract variability and function. The identification of CAG tract – containing ncRNAs, including disease-relevant circRNAs detectable in human tissues, points to their functional potential in the brain. The presence of circ1 at substantial levels in blood and cerebellum highlights the potential for further investigation of this circRNA, for example, in EVs isolated from blood or specific brain cell types. Overall, our results underscore the need to systematically investigate repeat-containing ncRNAs as active modulators of cellular pathways.

## Supplementary Material

Supplementary Tables and Text.docx

Table_S1.xlsx

Table_S4.xlsx

Table_S3.xlsx

Supplementary Figures.pdf

## Data Availability

The authors confirm that the data supporting the findings of this study are available within the article and its supplementary materials.
