# Supplementary material for: Discovery of a mutation-containing circRNA in polyglutamine disease through systematic analysis of RNAs with CAG repeats
Source: RNA Biol. 2026 Jun 24;23(1):1–12. doi: 10.1080/15476286.2026.2684791 (PMC13313202; doi:10.1080/15476286.2026.2684791)
Supplement: Supplementary Tables and Text.docx [file KRNB_A_2684791_SM1281.docx]

**SUPPLEMENTARY TABLES**

**Supplementary Table 1. Lists with information about identified mRNAs, lncRNAs, and circRNAs containing a CAG tract composed of at least 10 units**

Lists with information about identified mRNAs, lncRNAs, and circRNAs containing a CAG tract composed of at least 10 units. GENCODE transcript annotation labels: ensembl_havana and ensembl_havana_tagene – manual curation HAVANA and automatic Ensembl annotation pipeline agree on the RNA sequence; havana and havana_tagene – transcript sequence obtained via manual curation; ensembl – annotated so far only by automatic Ensembl annotation pipeline.

**Supplementary Table 2. Sequences of primers used in PCRs**

| Name of primer | Sequence 5’-3’ |
| --- | --- |
| ATXN7_circ1_F | AGGCTTCCAAACTTCCTGGG |
| ATXN7_circ1_R | CTTTCCGCTCCTTCCCGAC |
| ATXN7_circ1_CAG_F | GGCTCTGTCGGGAAGGAGC |
| ATXN7_circ1_CAG_R | CACTTCAGGACTGGGCAGAG |
| ATXN7_circ1_F2 | GACGGGACAGAATTGGACGA |
| HIPK3_circ_F | GTCGGCCAGTCATGTATCAA |
| HIPK3_circ_R | ACCAAGACTTGTGAGGCCAT |
| N4BP2L2_circ_F | CAAAGACCTCCTCCTCCACA |
| N4BP2L2_circ_R | TCAGTGCTGAACACAATGCC |
| ATXN7_F | GCCAGCTTCTCGGTTATCCA |
| ATXN7_R | TCCCAAATGTGCAAAAAGATGCT |
| GAPDH_F | GAAGGTGAAGGTCGGAGTC |
| GAPDH_R | GAAGATGGTGATGGGATTTC |
| EEF2_F | TCATCGAGGAGTCGGGAGAG |
| EEF2_R | ACGACCGGGTCAGATTTCTTG |
| MALAT1_F | GAATTGCGTCATTTAAAGCCTAGTT |
| MALAT1_R | GTTTCATCCTACCACTCCCAATTAAT |
| HPRT_F | TGACCTTGATTTATTTTGCATAC |
| HPRT_R | CGAGCAAGACGTTCAGTCCT |
| pre-ATXN7_F | CTGAGCCAGCTTCTCGGTTA |
| pre-ATXN7_R | GGTGGACGTGGAACTTACAGA |

**Supplementary Table 3. Predicted miRNA binding sites in circ1**

The table lists all miRNAs predicted to interact with circ1, as retrieved from the circAtlas database v. 3.0^1^. For each miRNA, the seed region sequence (positions 2–8 from the 5' end), the full mature miRNA sequence, the seed match type(s) identified in the circRNA sequence (8mer, 7mer-m8, 7mer-A1, 6mer, or no match), the total number of predicted binding sites, and the miRBase accession number (MIMAT) are provided. Mature miRNA sequences were retrieved from miRBase^2^ (release 22).

**Supplementary Table 4**. **Predicted RBP binding motifs in circ1**

The table lists all RNA-binding protein (RBP) binding motifs identified in the circ1 sequence. RBPs predicted to interact with circ1 were retrieved from the circAtlas^1^ and CircPedia^3^ databases. For each entry, the following information is provided: the RBP gene name, the motif sequence, the number of occurrences in the circRNA sequence, a description of the experiment in which the motif was determined, the experimental method used, and the ATtRACT confidence score. Motif sequences were retrieved from the ATtRACT database^4^ (*Homo* *sapiens* entries) and searched against the circRNA sequence using exact string matching. Two asterisks indicate experimentally validated motifs.

**SUPPLEMENTARY TEXT**

**Supplementary Methods**

**Details of retrieval of coding and ncRNAs with CAG tracts from databases**

Protein-coding transcripts and lncRNAs were retrieved from GENCODE release 49^5^ (September 2025), which was built on the GRCh38.p14 genome assembly. For protein-coding transcripts, the GENCODE 'protein_coding' biotype was utilized. This dataset consists of transcript models identified *via* HAVANA manual curation and the Ensembl GeneBuild automated annotation pipeline. Protein-coding sequences (CDS) together with their respective 5’ and 3’ untranslated regions (UTRs), excluding intronic sequences were analyzed. For lncRNAs, the GENCODE 'lncRNA' biotype (defined as non-coding transcripts >200 bp) was used; this set incorporates RNAs mapped through HAVANA manual curation, Ensembl automated annotation, and the integration of capture long-read RNA sequencing (CLS) data^6^. Human circular RNA sequences identified, filtered and classified by circAtlas 3.0^1^ was used as circRNA dataset for the analysis. An in-house Python script was employed to identify RNAs containing a minimum threshold of 10 consecutive CAG repeats among protein-coding transcripts, lncRNAs, and circRNAs, as previously described^7^. No additional post-hoc filtering steps were applied. Following the identification of protein-coding transcripts and lncRNAs containing CAG repeat tracts, the GENCODE transcript source (HAVANA vs. Ensembl) was retrieved using Ensembl BioMart. For circRNAs, identifiers were standardized according to recent nomenclature guidelines^8^, and cross-annotations were integrated from CIRCpedia v2^9^, CIRCpedia v3^3^, deepBase v2.0^10^, circBase^11^, and circRNADb^12^. All identified RNAs are provided in Supplementary Table S2.

**CAG tract polymorphism analysis**

Population-scale data regarding specific *loci* were derived from TR Atlas^13^ (<https://wlcb.oit.uci.edu/TRatlas>) that combines information from main populations: European, Hispanic, East Asian, South Asian and African. Additionally, the database also includes information about sub-populations.

**Data retrieval and analysis of the relationship between CAG tracts and distance from the back-splice junction (BSJ)**

CircRNA sequences were retrieved, and consecutive CAG trinucleotide repeats were identified in R (version 4.5.1; R Core Team, 2025). For each repeat, the distance to the nearest BSJ was calculated as the minimum of the distance to the sequence start or end, reflecting the circular topology of the molecule. The distribution of distances was visualized as a kernel density estimate using the default Gaussian kernel in ggplot2.

**Identification of miRNA and RBP binding sites**

To identify putative binding sites for RNA-binding proteins (RBPs) and microRNAs (miRNAs) within the circ1 sequence, sequence-based analyses were performed using custom R scripts (version 4.5.1; R Core Team, 2025) with the Biostrings and tidyverse packages. Lists of RBPs and miRNAs predicted to interact with circ1 were obtained from the circAtlas v3.0^1^ and CircPedia v3^3^ databases. Known RBP binding motifs were retrieved from the ATtRACT database v0.99β^4^ (*Homo sapiens* entries) and searched against the circRNA sequence using exact string matching; only motifs with at least one occurrence were retained. Mature miRNA sequences were retrieved from miRBase^2^ (release 22). For each miRNA, binding sites were identified based on complementarity between the seed region (positions 2–8 from the 5' end) and the circRNA sequence, following the TargetScan^14^ nomenclature: 8mer (perfect match at positions 2–8 with an adenosine at target position 1), 7mer-m8 (perfect match at positions 2–8), 7mer-A1 (perfect match at positions 2–7 with an adenosine at target position 1), and 6mer (match at positions 2–7).

**Cell culture**

Human fibroblasts were cultured in MEM (Gibco), supplemented with 10% fetal bovine serum (EurX), 100 U/ml penicillin–streptomycin (Gibco), and 2 mM L-glutamine (Gibco), and passaged using trypsin. HEK293T cells (ATCC) were cultured in DMEM (Gibco) with the same supplements. NSCs were obtained by iPSC differentiation using STEMdiff SMADi Neural Induction Kit (STEMCELL Technologies) and a monolayer protocol following the manufacturer’s instructions. After the third passage, NSCs were grown in STEMdiff Neural Progenitor Medium (NPM) (STEMCELL Technologies). NSCs were detached using StemPro Accutase Cell Dissociation Reagent (Gibco), seeded on Geltrex-coated (Gibco) plates and supplemented with 10nM Y-27632 (ROCK inhibitor) for 24h after each passage.

**RNA from human brain and liver**

Total RNA from human brain (First Choice Human Brain Total RNA) and liver (First Choice Human Liver Total RNA) was purchased from ThermoFisher.

Bulk RNA was purified from post-mortem human cerebellar tissues by pulverizing tissue with a cold mortar and pestle on dry ice. 50 mg of pulverized tissue was then homogenized in 500 µL TRIzol (ThermoFisher) and RNA was purified with the Direct-zol RNA MiniPrep Plus kit (Zymo Research) or RNeasy Plus Kit (Qiagen). Total RNA was then treated with the TURBO DNA-free kit (ThermoFisher).

**RNA isolation from cell lines**

Total RNA was isolated from cell pellets with TRI Reagent (Invitrogen) using the Total RNA Zol-Out D kit (A&A Biotechnology). Briefly, samples in TRI Reagent were mixed with 1 volume of 100% ethanol and loaded onto microcolumns. Following RNA binding, on-column DNase digestion was performed for 30 min at 37 °C. RNA was eluted in RNase-free water after a short incubation at room temperature. RNA concentration was determined using a spectrophotometer (DeNovix).

**RNA isolation from blood**

Total RNA was isolated from peripheral whole blood using the manual protocol of the PAXgene Blood RNA Kit (PreAnalytiX), according to the manufacturer’s instructions. Following collection, tubes were incubated for 2h at room temperature to ensure complete lysis of blood cells and stored at –80 °C until extraction. Briefly, frozen samples were thawed and centrifuged, the supernatant was removed, and the pellet was washed with RNase-free water. After a second centrifugation, the pellet was resuspended and incubated in buffer with proteinase K. Lysates were homogenized by centrifugation through the PAXgene Shredder spin column, and the cleared flow-through was mixed with ethanol and loaded onto PAXgene RNA spin columns. After binding, RNA was purified through sequential washes. Between the first and second wash steps, the membrane was treated with DNase I to remove trace DNA. RNA was eluted in elution buffer, heat-denatured, and quantified using a Qubit 4 fluorometer with the Qubit RNA BR Assay kit (Invitrogen). RNA integrity was assessed using the 4150 TapeStation system (Agilent).

**RNase R treatment**

1 µg of total RNA was treated with 4U of RNase R (BioVision, Inc.) for 30 min at room temperature. The reaction was terminated by adding RNase OUT (Invitrogen) and subsequently purified using acid phenol:chloroform, following the TRIzol Reagent protocol from the phase separation step onward. To remove residual organic contaminants, the RNA was further purified by precipitation with 1/10 volume of 3 M sodium acetate at –80°C for 30 min. Samples were centrifuged at 10,400 x *g* for 15 min, and the resulting pellets were washed with 1 ml of 75% cold ethanol, followed by centrifugation for 10 min. Pellets were air-dried and resuspended in nuclease-free water. RNA concentration and purity were assessed using a DeNovix spectrophotometer.

**Polysome profiling**

Polysome profiling was performed using 10-60% sucrose gradients prepared with the Gradient Station (BIOCOMP) in buffer containing 100 mM KCl, 20 mM HEPES (pH 7.6), 5 mM MgCl_2_, 100 µg/ml cycloheximide, 5 U/ml RNase inhibitor (Applied Biosystems), and 1x Protease Inhibitor Cocktail EDTA-free. Gradients were prepared and cooled prior to use.

Cycloheximide was added to the culture medium to a final concentration of 100 µg/ml, and cells were incubated for 5 minutes at 37°C. Cells were then washed with 4 ml of ice-cold PBS containing 100 µg/ml cycloheximide. HEK293T cells were scraped on ice in 1,5 ml ice-cold PBS with 100 µg/ml cycloheximide. NSCs were detached using StemPro Accutase Cell Dissociation Reagent (Gibco).

Cells were collected by centrifugation at 10 x *g* (HEK293T) or 270 x *g* (NSCs) for 5 minutes at 4°C. Pellets were resuspended in 500 µl ice-cold lysis buffer (10 mM HEPES pH 7,9; 1,5 mM MgCl_2_; 10 mM KCl; 0,5 mM DTT; 1% CHAPS; 100 U/ml RNase inhibitor; 100 µg/ml cycloheximide) and incubated for 10 min on ice. Lysates were centrifuged at 1500 x *g* for 5 minutes at 4°C. The absorbance of 100x diluted lysates was measured at 260 nm, and lysates containing 24 OD (for HEK293T) and 3 OD (for NSCs) were loaded onto previously prepared sucrose gradients. Gradients were centrifuged at 39 000 rpm for 2 hours and 40 minutes at 4°C using the SW 41Ti rotor (Beckman Coulter). 30 fractions were collected using The Gradient Station (BIOCOMP). Then each collected fraction was mixed with an equal volume of TRI Reagent (Invitrogen).

Total RNA was isolated using the Total RNA Zol-Out D kit (A&A Biotechnology). Fractions were assigned to ribonucleoproteins (RNPs), 40S, 60S, 80S or polysomes according to the obtained A260 graphs and agarose gel resolution of collected fractions. Then, equal volumes of total RNA samples were mixed in case more than one fraction was assigned to a group. The abundance of each transcript in a given fraction was expressed as a percentage of the total signal across all fractions. Cq values were first converted to a linear scale using the formula 2^(40−Cq), and the fractional abundance was calculated as:

$$\mathrm{Enrichment}\left( \% \right)= 100 x\frac{2^{40-\mathrm{Cq}_{i}}}{\sum_{i=1}^{n} 2^{40-\mathrm{Cq}_{i}}}$$

where Cqᵢ is the quantification cycle value for fraction i, and the sum encompasses all analyzed fractions (i = 1 to n).

**Nuclear and cytoplasmic fractionation**

Fibroblast cells were fractionated using NE-PER Nuclear and Cytoplasmic Extraction Reagents (Thermo Scientific) according to the manufacturer’s protocol. Briefly, cells were collected and resuspended in CER I buffer supplied with RNaseOUT RNase Inhibitor (Invitrogen) and Halt Protease Inhibitor Cocktail (Thermo Scientific). After 10 min incubation on ice, CER II buffer was added, followed by an additional 9 min incubation on ice. Samples were then centrifuged at 16 000 x *g* to separate cytoplasmic supernatant from the nuclear pellet. Both fractions were mixed with 500 μL of TRI Reagent, and RNA was isolated using the phenol-chloroform extraction method. DNase treatment was performed using the TURBO DNA-free Kit according to the manufacturer’s instructions. Equal volumes of RNA from each fraction were used for reverse transcription. The percentage of transcript localized in the nucleus was calculated as the percentage of nuclear signal relative to the total signal from nuclear and cytoplasmic fractions, using the equation:

$$Nuclear distribution=100\% \times\frac{2^{-{Cq}_{nuc}}}{2^{-{Cq}_{nuc}}+ 2^{-{Cq}_{cyto}}}$$

Similarly, the percentage of transcript in the cytosol was calculated using:

$$Cytosol distribution=100\% \times\frac{2^{-{Cq}_{cyto}}}{2^{-{Cq}_{nuc}}+ 2^{-{Cq}_{cyto}}}$$

*MALAT1* and *HPRT* were included as controls for nuclear and cytoplasmic localization, respectively.

**Secondary RNA structure modeling**

Secondary structures of circ1 were predicted using RNAfold webserver^15–17^, using default parameters (with option selected: “assume RNA molecule to be circular”). Minimal free energy (MFE) secondary structures are presented in Fig. 2F with base-pair probabilities indicated for each nucleotide according to colors shown in the legend (0 to 1 scale indicates the probability for pairing or remaining unpaired).

**Supplementary References**

1. Wu, W., Zhao, F. & Zhang, J. circAtlas 3.0: a gateway to 3 million curated vertebrate circular RNAs based on a standardized nomenclature scheme. *Nucleic Acids Res.* 52, D52–D60 (2024).

2. Kozomara, A., Birgaoanu, M. & Griffiths-Jones, S. miRBase: from microRNA sequences to function. *Nucleic Acids Res.* 47, D155–D162 (2019).

3. Zhai, S.-N. *et al.* CIRCpedia v3: an interactive database for circular RNA characterization and functional exploration. *Nucleic Acids Res.* 54, D78–D88 (2026).

4. Giudice, G., Sánchez-Cabo, F., Torroja, C. & Lara-Pezzi, E. ATtRACT-a database of RNA-binding proteins and associated motifs. *Database (Oxford).* 2016, (2016).

5. Mudge, J. M. *et al.* GENCODE 2025: reference gene annotation for human and mouse. *Nucleic Acids Res.* 53, D966–D975 (2025).

6. Perteghella, T. *et al.* GENCODE: massively expanding the lncRNA catalog through capture long-read RNA sequencing. *bioRxiv* https://doi.org/10.1101/2024.10.29.620654 (2024) doi:10.1101/2024.10.29.620654.

7. Witkos, T. M., Krzyzosiak, W. J., Fiszer, A. & Koscianska, E. A potential role of extended simple sequence repeats in competing endogenous RNA crosstalk. *RNA Biol.* 15, 1399–1409 (2018).

8. Chen, L. L. *et al.* A guide to naming eukaryotic circular RNAs. *Nat. Cell Biol.* 25, 1–5 (2023).

9. Dong, R., Ma, X. K., Li, G. W. & Yang, L. CIRCpedia v2: An Updated Database for Comprehensive Circular RNA Annotation and Expression Comparison. *Genomics Proteomics Bioinformatics* 16, 226–233 (2018).

10. Zheng, L. L. *et al.* deepBase v2.0: identification, expression, evolution and function of small RNAs, LncRNAs and circular RNAs from deep-sequencing data. *Nucleic Acids Res.* 44, D196–D202 (2016).

11. Glažar, P., Papavasileiou, P. & Rajewsky, N. circBase: a database for circular RNAs. *RNA* 20, 1666–1670 (2014).

12. Chen, X. *et al.* circRNADb: A comprehensive database for human circular RNAs with protein-coding annotations. *Sci. Rep.* 6, (2016).

13. Cui, Y. *et al.* A genome-wide spectrum of tandem repeat expansions in 338,963 humans. *Cell* 187, 2336-2341.e5 (2024).

14. McGeary, S. E. *et al.* The biochemical basis of microRNA targeting efficacy. *Science* 366, (2019).

15. Lorenz, R. *et al.* ViennaRNA Package 2.0. *Algorithms Mol. Biol.* 6, (2011).

16. Mathews, D. H. *et al.* Incorporating chemical modification constraints into a dynamic programming algorithm for prediction of RNA secondary structure. *Proc. Natl. Acad. Sci. U. S. A.* 101, 7287–7292 (2004).

17. Gruber, A. R., Lorenz, R., Bernhart, S. H., Neuböck, R. & Hofacker, I. L. The Vienna RNA websuite. *Nucleic Acids Res.* 36, (2008).
