# Supplementary material for: Discovery of a mutation-containing circRNA in polyglutamine disease through systematic analysis of RNAs with CAG repeats
Source: RNA Biol. 2026 Jun 24;23(1):1–12. doi: 10.1080/15476286.2026.2684791 (PMC13313202; doi:10.1080/15476286.2026.2684791)
Supplement: Supplementary Figures.pdf [file KRNB_A_2684791_SM1277.pdf]

SUPPLEMENTARY FIGURES

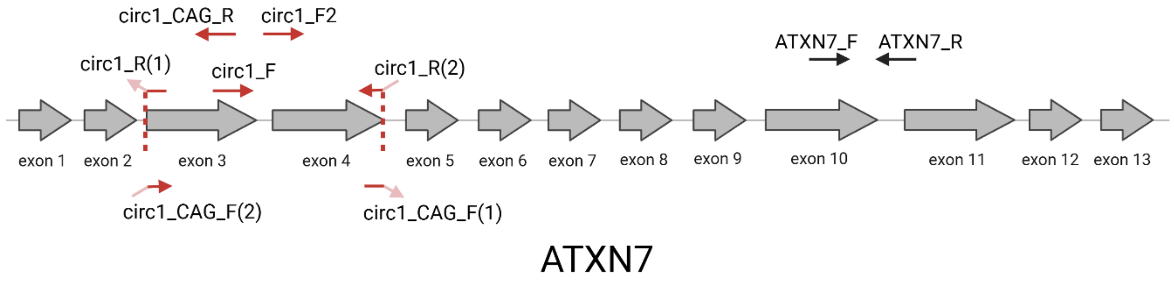

Supplementary Figure 1. Graphical representation of exons in the *ATXN7* locus.
Arrows indicate the locations of primers used for RT-PCR: red for circ1 and black for the linear transcript. The circ1_R and circ1_CAG_F primers are shown in two parts, as they span the back-splicing junction between exons 3 and 4, indicated by the dashed red line. The presented lengths of the exons do not reflect the actual lengths of *ATXN7* exons. The primer pairs used for specific results generation are listed.
circ1_F & circ1_R – Fig. 2B-D, 3A, 3C, S4C, S5B, S5C, S7B, S9.
circ1_CAG_F & circ1_CAG_R – Fig. 2A, 2E, 3B, 3D.
circ1_F2 & circ1_CAG_R – Fig. S5A.
ATXN7_F & ATXN7_R – Fig. 2B-D, 3A, 3C, S4C, S5B, S5C, S7B, S9.

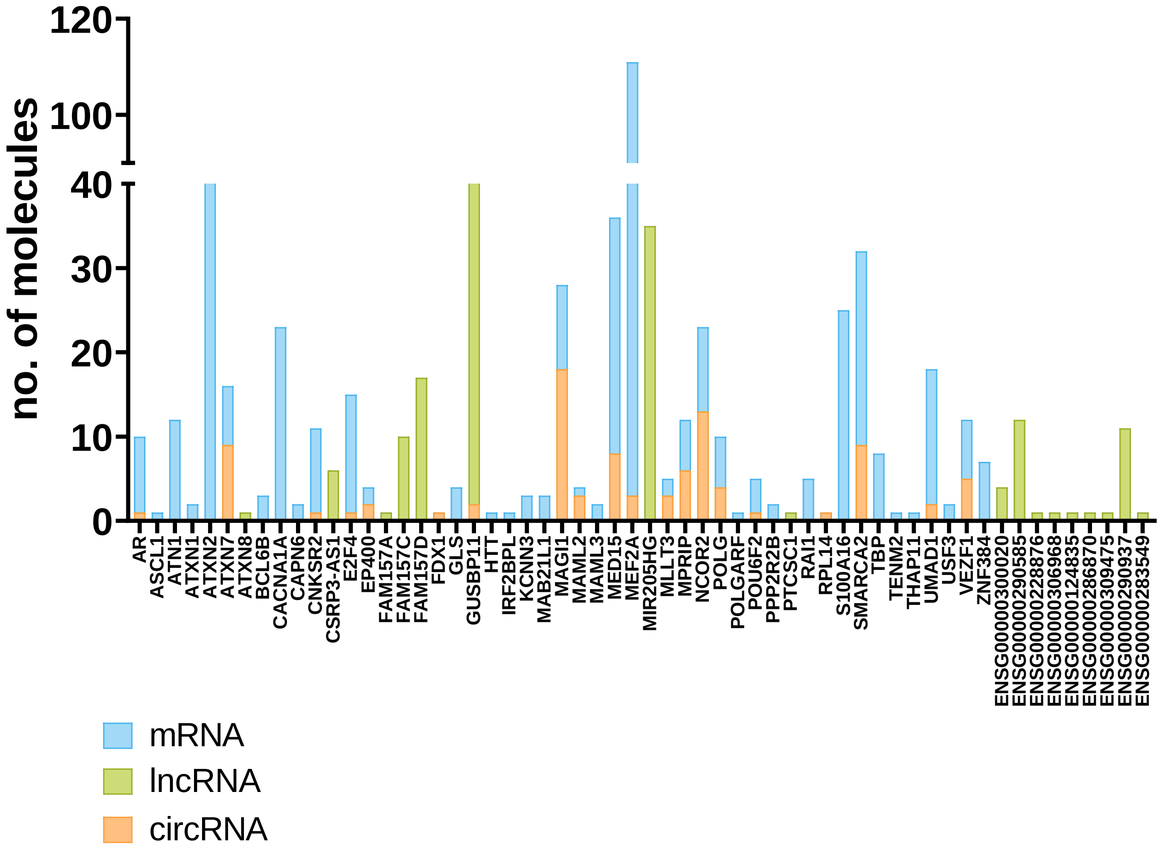

Supplementary Figure 2. The number of variants of CAG-containing RNA molecules across 58 genomic loci and 3 RNA biotypes

The number of mRNA (blue), lncRNA (green), and circRNA (orange) molecules for each genomic locus is presented.

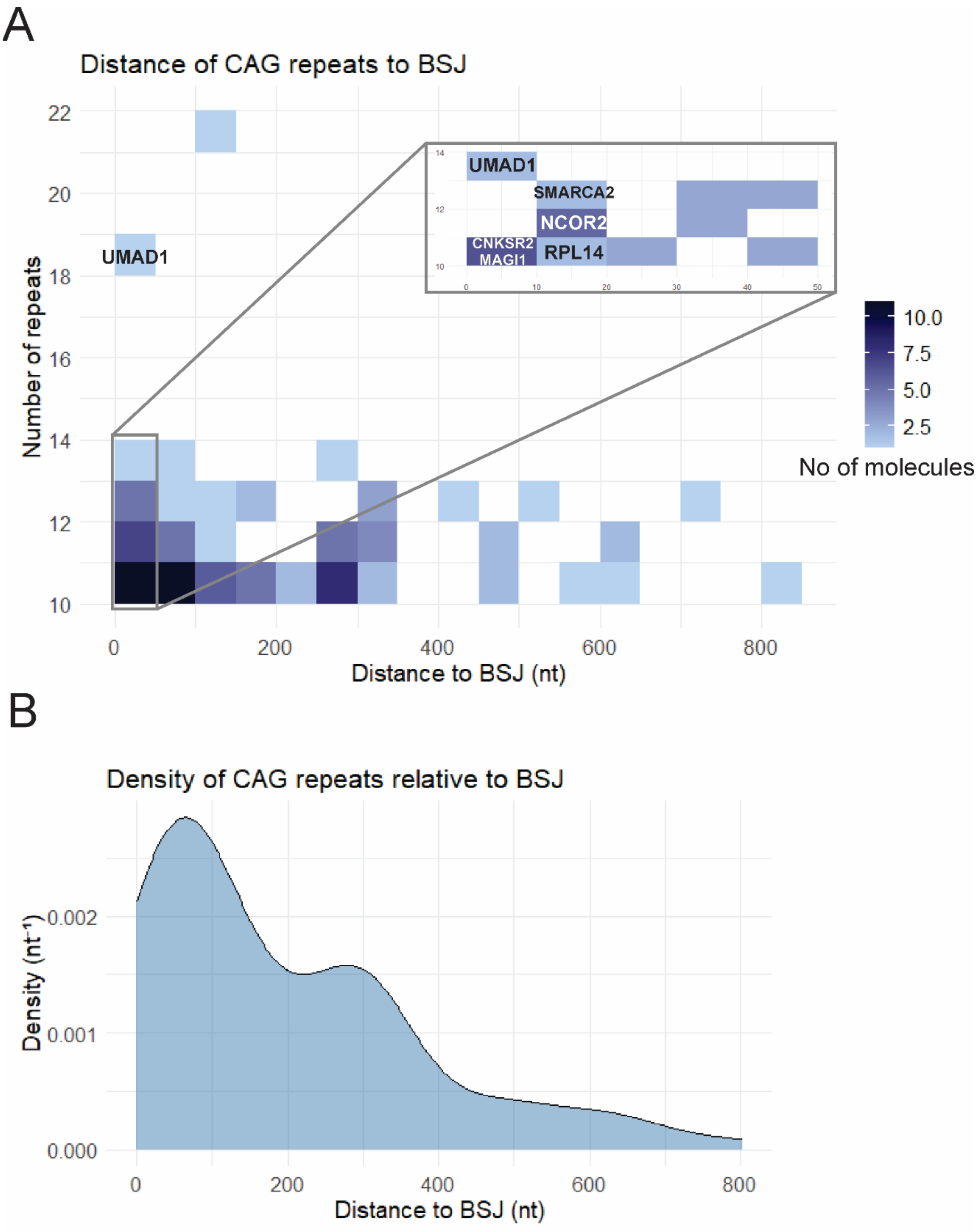

Supplementary Figure 3. Distance of CAG repeat tracts in circRNA sequences relative to the back-splice junction (BSJ)

A Heatmap showing the relationship between the number of consecutive CAG repeats (≥10) in circRNAs and the distance of the tract to the BSJ (bin size: 50 nt × 1 repeat). The inset shows sequences within 50 nt of the BSJ at higher resolution (bin size: 10 nt × 1 repeat). Selected circRNAs are labeled with the locus name. Color scale indicates number of circRNA molecules per bin.

B Kernel density estimate of CAG repeat distance to the BSJ. The Y-axis represents probability density; the area under the curve sums to 1.

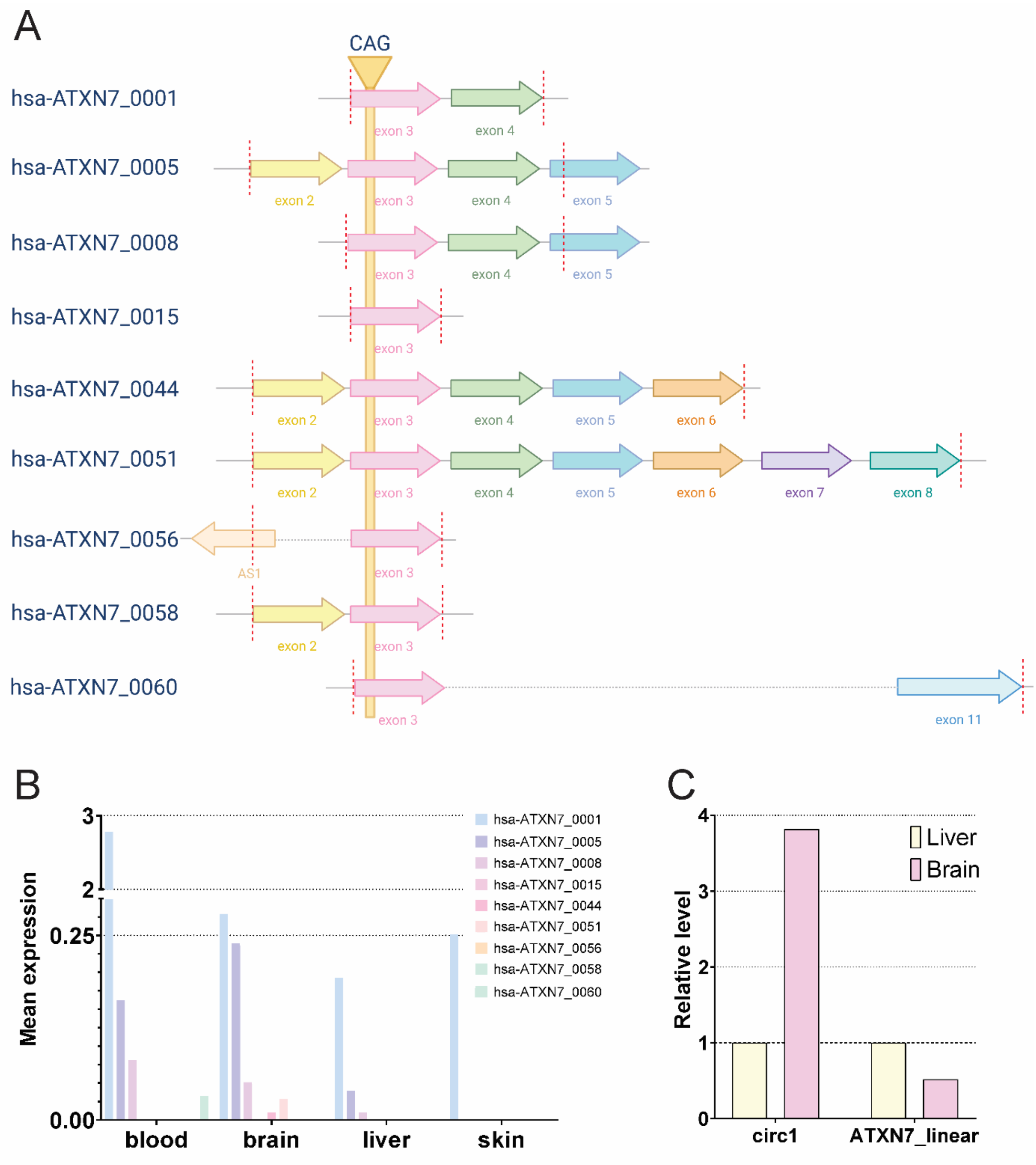

Supplementary Figure 4. Characteristics of circRNAs originating from *ATXN7* locus

A Graphic representation of exon and intron composition of CAG repeat-containing circRNA molecules from the *ATXN7* locus. The dashed red line indicates the back-splice junction site. The grey dotted line indicates an intronic sequence. hsa-ATXN7_001 is circATXN7(3,4).1; named hereafter "circ1".

B Mean expression levels of circRNAs from the *ATXN7* locus in selected tissues derived from the circAtlas reference dataset.

C The relative level of circ1 and *ATXN7* mRNA (ATXN7_linear) in commercially available human tissue lysates was assessed using RT-qPCR. For normalization, the mean results from liver tissue were set as 1 and used as a reference for brain tissue. Results for circ1 represent the circ/linear RNA ratio calculated using linear RNA Ct as a reference; *GAPDH* and *EEF2* were used as references for ATXN7_linear.

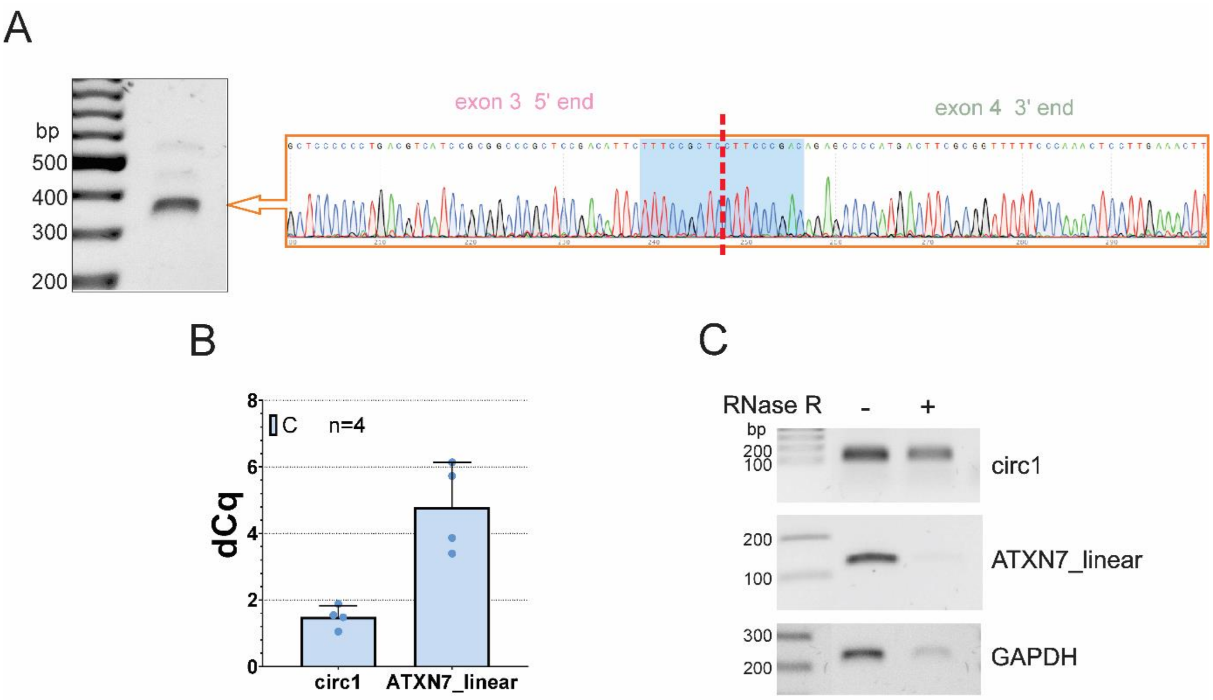

Supplementary Figure 5. Circular nature confirmation of circ1 in human fibroblasts

A RT-PCR product for circ1 obtained with divergent primers from RNA isolated from the control line and Sanger sequencing results showing back-splice junction (BSJ), indicated by a dashed red line. Expected amplicon size: 359 bp.

B The difference in quantification cycle [dCq = Cq (RNase R+) – Cq (RNase R-)] values of circ1 and linear *ATXN7* mRNA (n = 4; C - GM07492) in a cell line samples without and after RNase R treatment was determined using RT-qPCR.

C RT-PCR product of circ1, ATXN7_linear and GAPDH amplification in fibroblast sample (GM07492) with and without RNase R treatment. Expected amplicon size: circ1 – 106 bp; ATXN7_linear – 122 bp; GAPDH – 226 bp.

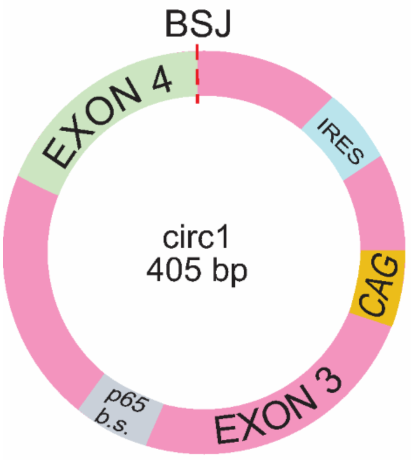

circ1 sequence (5' – 3')

GAGCGGAAAGAAUGUCGGAGCGGGCCGCGGAUGACGUCAGGGG
GGAGCCGCGCCGCGCGGCGGCGGCGGCGGGCGGAGCAGCGGCCG
CGGCCGCCCGGCAGCAGCAGCAGCAGCAGCAGCAGCAGCAGCCGC
CGCCUCCGCAGCCCCAGCGGCAGCAGCACCCGCCACCGCCGCCACG
GCGCACACGGCCGGAGGACGGCGGGCCCGGCGCCGCCUCCACCUC
GGCCGCCGCAAUGGCGACGGUCGGGGAGCGCAGGCCUCUGCCCA
GUCCUGAAGUGAUGCUGGGACAGUCGUGGAAUCUGUGGG**UUG**A
GGCUUCCAAACUUCCUGGGAAGGACGGGACAGAAUUGGACGAAA
GUUUCAAGGAGUUUGGGAAAAACCGCGAAGUCAUGGGGCUCUG
UCGGGAAG

Potential peptide sequence:

LRLPNFLGRTGQNWTKVSRSLGKTAKSWGSVGKERKECRSGPRMTSG
GSRAARRRRRAEQRPRPPGSSSSSSSSSSSRRLRSPSGSSTRHRRHGAHG
RRTAGPAPPPPRPPQWRRSGSAGLCPVLK

Supplementary Figure 6. Predicted translation of circ1

Schematic representation of circ1 with predicted internal ribosome entry site (IRES) indicated. The full nucleotide sequence of circ1 is shown to the right, with the IRES sequence highlighted in blue, the start codon bolded, and the potential open reading frame (ORF) underlined. Below the sequence, the potential peptide sequence encoded by circ1 is provided.

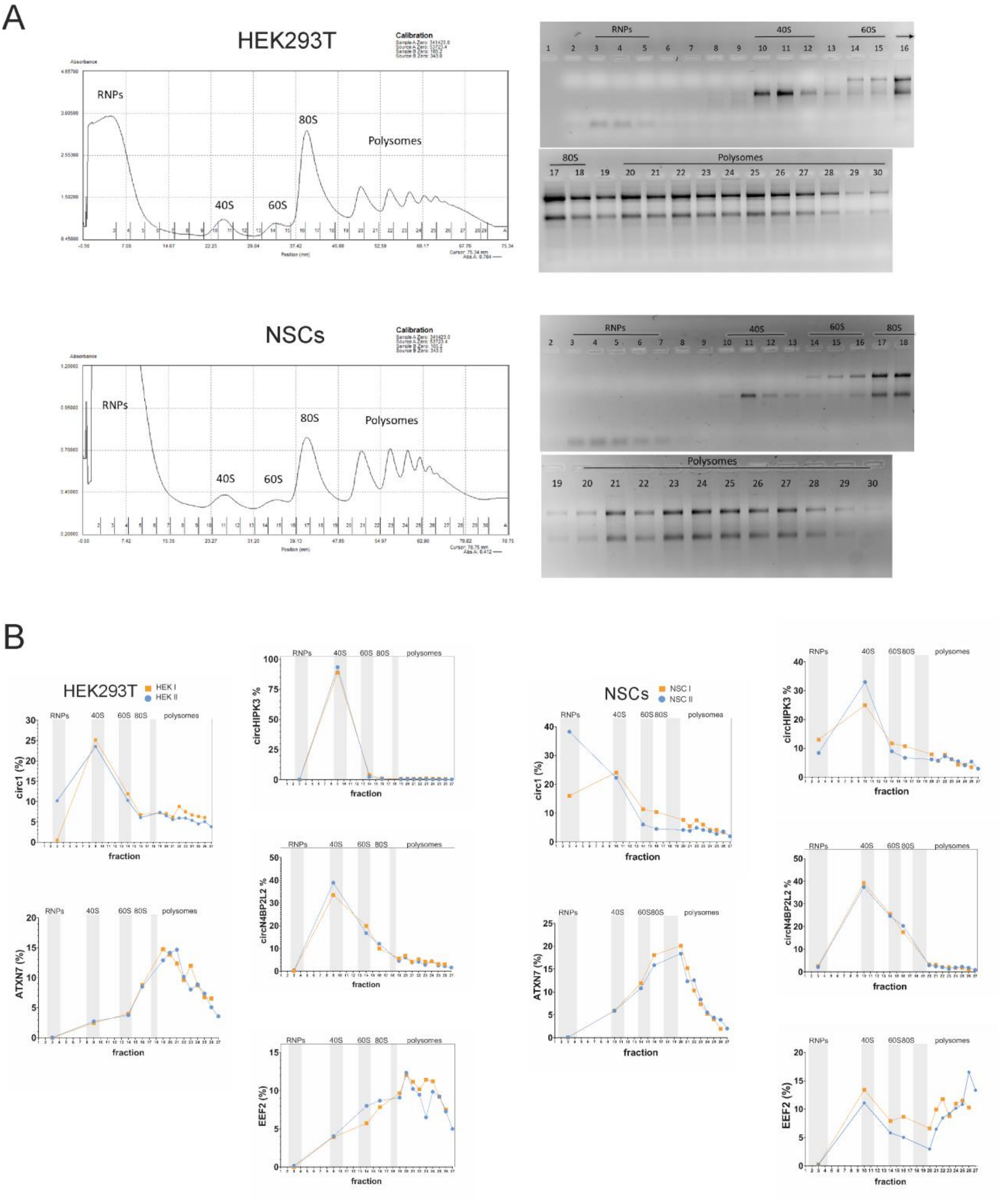

Supplementary Figure 7. Additional results for polysome fractionation

A The representative sucrose gradient A260 absorbance profiles of polysome fractionation of lysates from HEK293T cells and NSCs (left panels) and representative agarose gel electrophoresis of total RNA isolated from each fraction (right panels). Numbers on gels indicate collected fractions.

B Distribution of circ1, *ATXN7,* circ_HIPK3, circ_N4BP2L2 and *EEF2* RNAs in fractions from HEK293T cells (left panel) and NSC (right panel), from two replicates of the experiment (marked in orange and blue). RNPs – ribonucleoproteins.

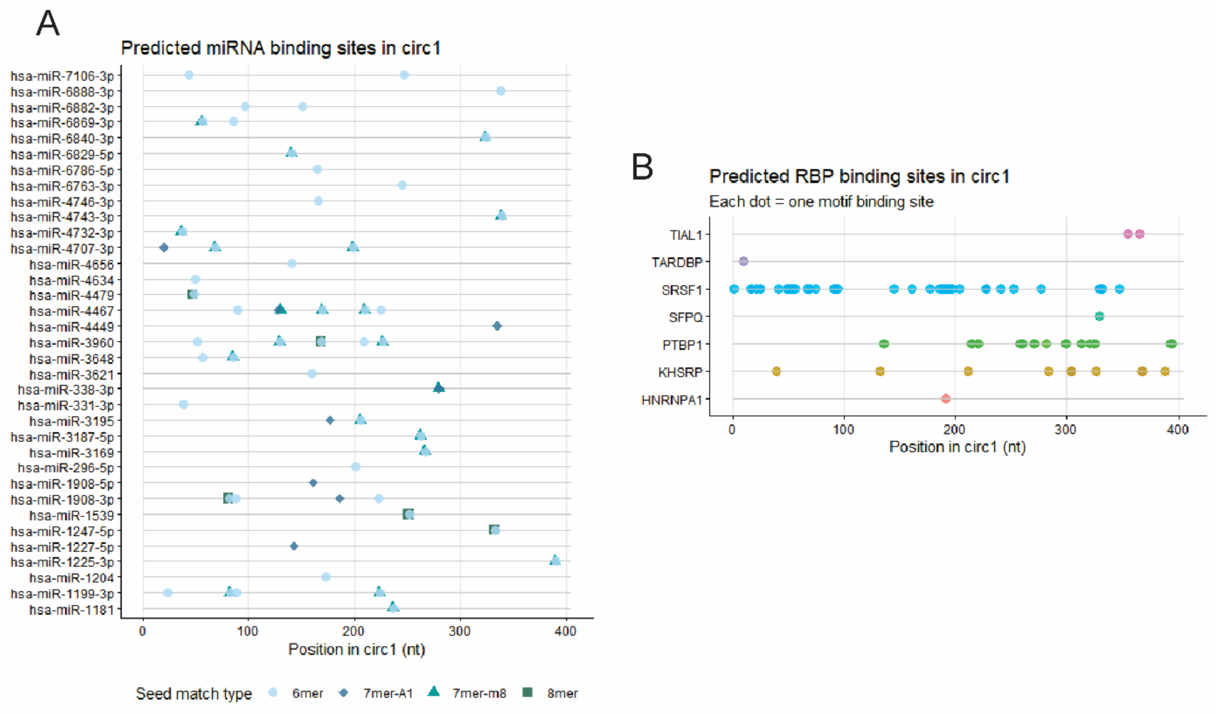

Supplementary Figure 8. Predicted miRNA- and protein- binding sites in circ1

A Predicted miRNA binding sites in circ1, based on circAtlas. Each point represents a single seed match site identified along the circRNA sequence. Binding sites were classified according to the TargetScan nomenclature based on complementarity between the miRNA seed region and the target sequence: 8mer — perfect match at positions 2–8 with an adenosine at target position 1 (strongest); 7mer-m8 — perfect match at positions 2–8; 7mer-A1 — perfect match at positions 2–7 with an adenosine at target position 1; 6mer — match at positions 2–7 (weakest). Mature miRNA sequences were retrieved from miRBase (release 22). Each miRNA is displayed on a separate track; horizontal lines represent the full length of circ1.

B Predicted RBP binding sites in circ1, based on circAtlas and CircPedia. Each dot represents a single motif match identified by exact sequence matching against the ATtRACT database (*Homo sapiens* entries; accessed April 3, 2026). Each RNA-binding protein (RBP) is displayed on a separate track along the full length of circ1. Horizontal lines represent the circ1 sequence; dot positions indicate the nucleotide coordinates of predicted binding sites.

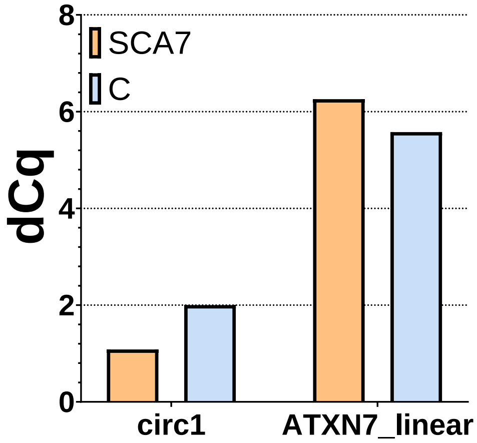

Supplementary Figure 9. The difference in quantification cycle (dCq) values of circ1 and linear *ATXN7* mRNA in blood samples after RNase R treatment was determined using RT-qPCR. C – healthy control; SCA7 – patient.
